# The Mount Vernon Hospital

**Published:** 1902-03-15

**Authors:** 


### THE MOUNT VERNON HOSPITAL.

### A YEAR OF PROGRESS.

Evidently the Governors of the Mount Vernon Hospital for Consumption, Hampstead and Northwood, as it is now to be called, are of a very trusting disposition, for they leave the annual meeting practically in the hands of their committee; there were perhaps four present on Wednesday afternoon last week who were not committee members, but certainly not more. Two of these were ladies.

The speech of Mr. Benjamin A. Lyon, chairman of the hospital, who has done so much to bring it up to its present high position among London hospitals, was undoubtedly the feature of the meeting. Mr. B. L. Cohen, M.P., occupied the chair, and the report being taken as read, Mr. Lyon said that during the eleven years that had elapsed since Mr. Cohen had acted as chairman the hospital had exactly doubled itself. Going back further still, to 1886, when Lord Knutsford took the chair at the festival dinner, Sir Sydney Waterlow scolded Mr. Lyon for having a dinner at all. "Our dinners," Mr. Lyon went on, "have made us."

#### Remarkable Generosity.

He had moved the adoption of the report for a great many years, but never had he done so under such good circumstances as to-day. The income had been over £10,000, and close upon £10,000 had been received towards the building fund, which required about £13,000 or £14,000, so that only some four or five thousand pounds had now to be made up. The hospital had found favour in the eyes of men of exceptional generosity, among whom was a man who wanted to build a convalescent home. He was one of the elect of the earth. He had given £121,000, which would leave after the purchase of the ground—which was already in possession—and the furnishing, laundry, etc., £50,000 for an endowment, and sufficient for the first four or five years, at the end of which time it was to be hoped the public would be so much attracted by the work that they would contribute largely. The building would practically double the accommodation. There would be 145 or 150 patients at Hampstead when the building was finished then, besides the 100 at Northwood—thus practically doubling the number they could take at present—and there would be a corresponding increase in staff. The Princess Christian had promised to lay the foundation stone at Northwood on May 13th. Her Royal Highness had visited the hospital, and had left happy memories of her kindness and graciousness. That, however, did not exhaust the list of what had been done.

New Out-Patients' Department.

The room in which the meeting was held was part of the out-patients' department at 41 Fitzroy Square. When the committee bought the freehold it was a quiet place, and very suitable for the purpose. Since, however, Messrs. Maple, Shoolbred, and Pickford had thought well to make the road a thoroughfare for their vans, the one quality for which it was valuable was destroyed, and the doctors complained that it was impossible to get a good diagnosis of the cases while there was so much noise. The committee had therefore been looking out for a more suitable place, and only within the last five or six weeks they had secured the house formerly belonging to Sir Charles Eastlake. This the committee had bought. In this house all the necessary departments could be arranged, and one room would be set apart for lectures to young medical students pursuing medical research in connection with tubercular disease, for which, hitherto, they had had to go elsewhere. The house was No. 7 in Fitzroy Square, so that the change of address would be simple. It was a building that would answer all the purposes of the committee, and it was absolutely quiet; the work of the out-patients' department would go on steadily when once they were installed there. Close upon 4,000 people came every year to consult the doctors, and got, if not a cure, at least a wonderful amount of relief—they were at any rate patched up for a time. These were people who would not go into the hospital—perhaps they could not; and for want of this treatment they would pass away and die. One word more. All this programme required money. Extraordinary gifts had been received, but more would be wanted, and he had already mentioned that a balance of £4,000 or £5,000 was still to be made up on the building fund. Then there was the purchase of the freehold of No. 7. At any rate, the change was one which he thought would commend itself to the governors.

That these remarks were received with equanimity was probably due to the fact that the committee knew them all before, and it seemed especially a pity, that after such a year's work, the whole body of governors was not present to give their commendation of the work done. "They know it's going on well," one of the committee remarked; "if there's going to be a row they come, but not when things are progressing satisfactorily."

